# Weak patriline effects are present in the cuticular hydrocarbon profiles of isolated *Formica exsecta* ants but they disappear in the colony environment

**DOI:** 10.1002/ece3.319

**Published:** 2012-08-14

**Authors:** Stephen Martin, Kalevi Trontti, Sue Shemilt, Falko Drijfhout, Roger Butlin, Duncan Jackson

**Affiliations:** 1 Department of Animal & Plant Sciences, University of SheffieldSheffield, S10 2TN, U.K; 2 Department of Biosciences, University of HelsinkiPO Box 65 (Viikinkaari 1), 00014, Helsinki, Finland; 3 Chemical Ecology Group, School of Physical and Geographical Sciences, Lennard-Jones Laboratory, Keele UniversityStaffordshire, ST5 5BG, U.K

**Keywords:** Alkanes, alkenes, ants, cuticular hydrocarbon, nepotism, patrilines, recognition

## Abstract

Chemical recognition cues are used to discriminate among species, con-specifics, and potentially between patrilines in social insect colonies. There is an ongoing debate about the possible persistence of patriline cues despite evidence for the mixing of colony odors via a “gestalt” mechanism in social insects, because patriline recognition could lead to nepotism. We analyzed the variation in recognition cues (cuticular hydrocarbons) with different mating frequencies or queen numbers in 688 *Formica exsecta* ants from 76 colonies. We found no increase in the profile variance as genetic diversity increased, indicating that patriline effects were absent or possibly obscured by a gestalt mechanism. We then demonstrated that an isolated individual's profile changed considerably relative to their colony profile, before stabilizing after 5 days. We used these isolated individuals to eliminate the masking effects of the gestalt mechanism, and we detected a weak but statistically significant patriline effect in isolated adult workers and also in newly emerged callow workers. Thus, our evidence suggests that genetic variation in the cuticular hydrocarbon profile of *F. exsecta* ants (*n*-alkanes and alkenes) resulted in differences among patrilines, but they were obscured in the colony environment, thereby avoiding costly nepotistic behaviors.

## Introduction

Recognition underpins all social interactions among organisms. Some mechanism of kin recognition, or perhaps kin/nonkin discrimination, is assumed to have been essential for the evolution of many forms of sociality, especially eusociality. To varying degrees, kin or nonkin might be detected based on the recognition or discrimination of phenotypes using genetically determined cues and/or familiar learned cues determined by shared environmental effects. Recognition of kin should enhance inclusive fitness by allowing an individual to direct altruistic acts toward its close kin, and this is a fundamental assumption of Hamilton's rule (Hamilton 1964). The ability to recognize kin is expected to be particularly important in social insects, because a colony can contain millions of related, nonreproductive workers whose only opportunity to increase their fitness is by favoring kin who share their genes. However, there is much debate over whether genetically determined kin recognition would be evolutionarily sustainable, primarily because it could lead to nepotism in the many insect societies that contain individuals with diverse genetic backgrounds (Keller 1997). Insect societies frequently contain queens that have mated with multiple males and or contain multiple queens. Thus, most researchers now acknowledge that recognition in social insect colonies is based on cues that facilitate the identification of other colony members, that is, nestmates, rather than evaluating genetic relatedness using kin-based cues (e.g., van Zweden et al. 2010).

Approximately 75% of social insect species produce colonies headed by a single queen (Hughes et al. 2008), but the queen typically mates with multiple males in many monogynous species to produce multiple patrilines within the same colony. This has allowed researchers to study the effects of different patrilines on behavior patterns, such as nepotism in honeybees (Châline et al. 2005a) and ants (Hannonen and Sundström 2003), worker policing in wasps (Bonckaert et al. 2008, 2011), and reproductive success in ants (Hughes and Boomsma 2008; Chéron et al. 2011). However, selection is predicted to act against genetic kin recognition cues. This is because clear kin recognition cues should lead to costly conflict between different patrilines where workers may try to ensure the propagation of their genes at the expense of other patrilines. Nepotistic behavior is assumed to be costly at the colony level (Keller et al. 1997), and accordingly no definitive evidence for nepotism has been forthcoming (Châline et al. 2005a; Zinck et al. 2009). Both this hypothesis and the available evidence suggest that genetically determined kin discrimination is unlikely to be found in social insects, while many consider that colony nestmate recognition in social insects is wholly mediated by environmentally acquired chemical cues that would overwhelm any genetic signals that might be exploited for nepotistic ends (Sorvari et al. 2008).

The major problem with this all-or-nothing approach to recognition is that these competing assumptions of recognition as either genetically or environmentally determined could engender a narrow view of what is evidently a complex system. Indeed, recognition cues must be genetically determined to some extent, because the biochemical production of chemical recognition cues is under genetic control (Morgan 2010). However, the subsequent mixing of these cues in a typically genetically heterogeneous environment will produce a colony profile that probably has to be learned from the social environment to identify nestmates at the colony level.

However, it is still argued that the characteristic split sex ratios found in some social insects, especially ants, must be driven by a relatedness asymmetry that requires workers to respond to the degree of genetic diversity in the colony (monandry vs. polyandry), which would be apparent as chemical differences among patrilines (Kümmerli and Keller 2009). Possible mechanisms of patriline discrimination have been identified by comparing whole cuticular hydrocarbon (CHC) profiles of a single honeybee (*Apis mellifera*) colony (Arnold et al. 1996), eight wood ant (*Formica truncorum*) colonies with multiply mated queens (Boomsma et al. 2003), four leaf-cutting ant (*Acromyrmex octospinosus*) colonies (Nehring et al. 2010), and three hornet (*Vespa crabro*) colonies (Dani et al. 2004). It was suggested that *n*-alkanes might encode kin recognition cues, mainly because these are the most abundant compounds and the easiest to detect using gas chromatography methods (Akino 2006). However, *n*-alkanes are unlikely to be used in patriline discrimination, because they are highly responsive to environmental factors such as temperature and humidity. The profiles of *n*-alkanes have been shown to be affected by worker tasks in honeybees (Kather et al. 2011) and ants, that is, foraging versus nonforaging (Wagner et al. 2001; Martin and Drijfhout 2009a), which would make them unreliable cues for patriline discrimination. Nehring et al. (2010) suggested that branched alkanes and alkenes could provide more patriline information than *n*-alkanes, which is more plausible given that only seven different *n*-alkanes are typically found in 78 ant species, whereas >1000 alkenes and branched *n*-alkanes are known (Martin and Drijfhout 2009b). Furthermore, the learning of *n*-alkanes and their differentiation is either lacking (Pickett et al. 1982; Sachse et al. 1999) or poor relative to other CHC groups (Châline et al. 2005b; Dani et al. 2005).

Nestmate recognition depends on the maintenance of different colony CHC profiles that are determined by genetic and environmental effects (Ozaki et al. 2005; Guerrieri et al. 2009). Genetic contributions to patriline-specific odors have been detected in bees (Greenberg 1979) and ants (Nehring et al. 2010), but there has been no demonstration that they can be exploited nepotistically. Only one previous study (Hannonen and Sundström 2003) has suggested that social insects workers favor their own close kin when rearing the brood, but a later study attributed this to variation in the viability of brood produced by different ant queens (Holzer et al. 2006). This failure to exploit kin recognition may be attributable to the homogenizing of colony CHC profiles via a colony “gestalt effect”, whereby social insects are suggested to produce a narrow colony profile via the exchange of CHCs during grooming (Soroker et al. 1995). This is hypothesized to result in a well-mixed colony CHC profile that eliminates any differences derived from genetic sources of variation within a colony, such as multiple mating, multiple queens, or adopted workers. However, the gestalt hypothesis may also eliminate any individual-specific signals that could be used to gauge relatedness between individuals, such as those detected in previous studies (Arnold et al. 1996; Boomsma et al. 2003; Nehring et al. 2010), provided that the rate of mixing exceeds that of CHC production. This would clearly negate the possibility of genetic kin recognition based on patrilines by ants that exhibit split sex ratios. To overcome this apparent contradiction, van Zweden et al. (2010) proposed that patriline signals might be encoded in *n*-alkanes, which they hypothesized would be less affected by mixing than other CHCs, such as alkenes, thereby allowing them to persist as a weak patriline signal. However, van Zweden et al. (2011) more recently showed that there was little evidence of heritability in the *n*-alkanes in the ant *Formica exsecta*, whereas there was greater evidence of heritability of a patriline signal in the alkenes. This signal was lost in colonies, presumably as a consequence of a gestalt mechanism.

**Figure 1:**
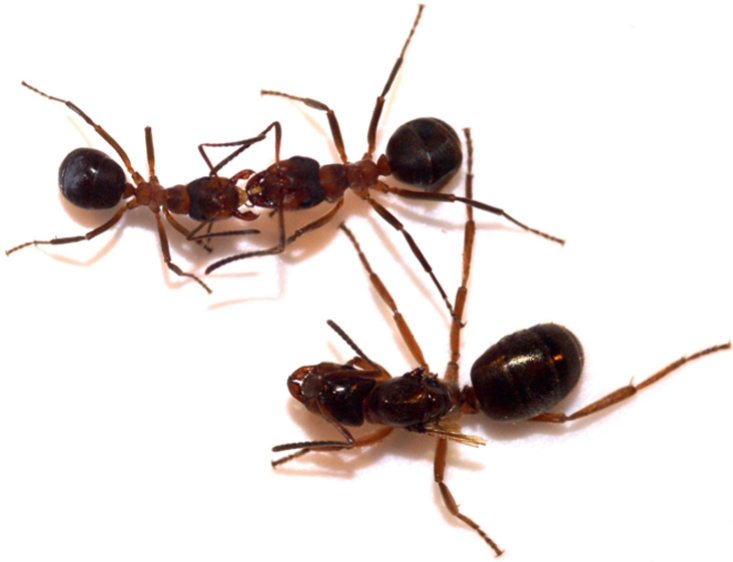
A *Formica exsecta* queen and two of her workers engaged in trophallaxis.

We aimed to resolve this issue by using two methods to determine the contribution of fathers to the within-colony variation of CHC profiles, that is, patriline effects, both under natural situations and when the gestalt effect was artificially removed by the isolation of callows (newly emerged workers) or adults from any colony effect. *Formica exsecta* (Fig. 1) has been an ideal model species for the study of nestmate recognition (Martin et al. 2008a,b), because its CHC profile is dominated by only 12 hydrocarbons (seven Z9-alkenes and five *n*-alkanes) with chain lengths from C_21_ to C_33_. It was previously demonstrated that the *n*-alkane composition and abundance varies with worker task in this species, that is, foraging or nonforaging (Martin and Drijfhout 2009a). However, three Z9-alkenes (C_23:1_, C_25:1_, and C_27:1_) dominate the alkene profile, where their amounts are always highly correlated among individuals within colonies (*r*^2^ > 0.97) and they provide a unique colony-specific profiles that are used for nestmate recognition (Martin et al. 2008a, 2011). These colony recognition profiles remain stable over years (Martin et al. 2012). In this species, pariline-specific Z9-alkene profiles were detected in only two of ten colonies studied by van Zweden et al. (2011) using PCA analysis performed on the z-transformed CHC data. *Formica exsecta* colonies contain a single queen (monogyny) or multiple queens (polygyny) that can each be mated with one (monandry), two, or three males (polyandry). In this study, the population consisted of up to 80 active colonies, which is a much larger scale of study than any previous research and this should ensure that artifacts due to small sample size are eliminated.

We tested three hypotheses: (1) increased genetic diversity leads to an increased variation in the colony profile; (2) an individual's profile becomes increasingly dissimilar from the colony profile during a period of isolation; and (3) isolated ants from different patrilines differ in their hydrocarbon profiles. We studied newly emerged callows because these are the final “brood” stage and the only stage of development that is not directly in contact with the colony, due to their protective cocoons. Therefore, they are the only individuals which are naturally isolated from any colony effects and studying them at emergence provided the best opportunity of detecting any differences in their CHC profiles with a genetic basis.

## Materials and Methods

### Study species and site

We studied the *F. exsecta* population on three islands (Joskär, Rovholmen, and Furuskär) in the Tvärminne archipelago, southwest Finland. This population has been subjected to a long-term study (Sundström et al. 2003; Vitikainen et al. 2011) since 1993, which has generated extensive behavioral and genetic data sets, including microsatellite and demographic data for about 150 colonies. Based on a hierarchical analysis, Sundström et al. (2003) found no significant genetic differentiation among these islands for either queens (F_pt_ = −0.005) or workers (F_pt_ = 0.001), but they did find a small significant differentiation among islands for colony fathers (F_pt_ = 0.023). This suggests sex-biased gene flow between islands in this *F. exsecta* population (Sundström et al. 2003). The population averages for within-colony relatedness in this population were *r* = 0.328 for seven polygynous colonies, *r* = 0.622 for 12 monogynous–polyandrous colonies, and *r* = 0.769 for 68 monogynous–monandrous colonies (Martin et al. 2011). In Finland, *F. exsecta* polygynous colonies appear to have been established from local monogynous colonies, followed by philopatric behavior and restricted gyne (new queen) emigration (Seppä et al. 2004).

In September 2008, ten worker ants from each of 85 colonies were collected from the surface of their mound. Ants were killed by freezing and then stored at −20°C, before analysis to determine the mean CHC profile for each colony. However, the collection method and storage conditions are not critical for insect CHCs, which remain stable for decades at ambient temperature (Martin et al. 2009a).

### Isolated adult workers

During September 2008, we collected a further 20 workers from each singly mated colony and 30 individuals from each doubly mated or polygynous colony. Each ant was picked from the nest mound using a thin piece of moss and placed into a 0.5 mL Eppendorf tube. This prevented the stressing of ants and avoided the release of formic acid, which results in the death of ants in the confines of the tube. There was a small air hole in each cap and tubes were maintained at 5°C. No food or water was provided. Tubes were checked daily until the ant died or moved slowly, at which point they were stored at −20°C prior to analysis. We have no evidence that this method significantly affected the Z9-alkene profile because live and dead nestmates isolated for the same period had the same Z9-alkene profiles (S. Martin and F. Drijfhout, unpubl. data), which is expected because the isolation of ants did not result in perturbed HC biosynthesis (Lenoir et al. 2001) although social isolation caused a progressive change in the overall CHC profile in the Lenoir et al. (2001) study. Isolation and starvation may cause stress and potentially affect the hydrocarbon production, but a consistent directional change would not produce variation among patrilines while an increase in variance would make patriline effects even more difficult to detect. Furthermore, it is very unlikely that the chemicals used for recognition would be susceptible to variation in conditions of stress, because any system that was highly responsive to environmental perturbations or individual status would perform poorly as a recognition medium.

### Isolated callow workers

In September 2009, we collected approximately 30 worker pupae from immediately beneath the mound's surface and 10 workers from the mound's surface from each of nine colonies. These pupae and workers were then maintained in individual Petri dishes in the laboratory at ambient temperature (15–24°C). Each isolated pupa was checked daily using a Leica (Leica Microsystems Ltd, Milton Keynes, Bucks, UK) binocular microscope (10×) equipped with cold light under-illumination. Callow workers were carefully released from their cocoons using forceps when fully developed, as indicated by pigmented eyes and leg movements. Each newly emerged ant was placed into a 0.5 mL Eppendorf tube and maintained at ambient temperature with no food or water. Each callow was checked daily until the ant died or moved slowly, at which point it was stored at −20°C prior to analysis.

### Chemical analysis

Each individual ant was placed in a glass vial with 50 μL of HPLC-grade hexane containing 1 mg 100 mL^−1^ of an internal standard (docosane, C_20_ alkane) for 10 min. The ant was removed and stored in ethanol at 5°C prior to genetic analysis, after the hexane extract was evaporated to dryness. Samples were analyzed on an Agilent 7890 gas chromatograph (GC) connected to an Agilent 5975 MSD (quadropole) mass spectrometer (MS; −70 eV, electron impact ionization) (Agilent Technologies, Berkshire, UK). The GC was equipped with a Zebron™ ZB-5HT column (length, 30 m; ID, 0.32 mm; film thickness, 0.25 μm), and the oven temperature was programmed from 50 to 110°C at 40°C min^−1^ and then from 110 to 360°C at 20°C min^−1^. Samples were injected in splitless mode, with helium as the carrier gas, at a constant flow rate of 1.0 mL min^−1^. Hydrocarbons were characterized using diagnostic ions and their Kovats indices. We rejected runs where the total ion count for all alkene ions did not exceed 10^7^, due to the difficulty of accurately integrating small peaks. Before chemical analysis commenced, five individual samples from five different *F. exsecta* colonies were run on a new high temperature column (maximum temperature 420°C), and it was confirmed that no additional hydrocarbons could be detected, that is, up to C_40_, compared with our previous analysis (Martin et al. 2008a).

### Genetic analysis

We sampled 20–40 individual ants (callows and adults) that had been isolated for at least 5 days, from colonies with known genetic characteristics, that is, monogynous or polyandrous. These ants were chemotyped (see above) and then genotyped at a diagnostic microsatellite locus to assign them to one of two known colony patrilines. Five additional colonies with unknown genetic profiles were sampled and screened using eight microsatellite markers (average heterozygosity H_E_ = 0.81) to assess the number of queens and matings. We genotyped 20–40 nonisolated adult workers from each of these colonies using the following loci: Fl21 (Chapuisat 1996); Fe13, Fe37, Fe38, Fe42, and Fe49 (Gyllenstrand et al. 2002); Fy3 (Hasegawa and Imai 2004); and Pla22 (Trontti et al. 2003), using the standard methods described in Vitikainen et al. (2011). Based on allele frequencies at these loci, this combination of microsatellites provided a nondetection error of <1‰, that is, the probability of an additional queen or male with a genotype identical to those observed.

### Data analysis

The peak area of each Z9-alkene and *n*-alkane was calculated by integrating the total ion chromatogram, for each individual sample. The Z9-alkene and *n*-alkane data were then analyzed separately, because previous studies have shown that these two classes of compounds have different functions (Martin and Drijfhout 2009a) and it has already been shown in this species that the Z9-alkenes alone have any power for separating patrilines (van Zweden et al. 2011). The relative amount (peak area) of each compound was log-contrast transformed (Aitchison 1986) using either C_25_ or C_25:1_ as a reference.


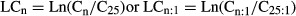


Log-contrasts were then standardized using the population mean and standard deviation (SD).

#### Hypothesis 1

##### Increased genetic diversity leads to increased variation in the profile

Estimates of the total within-colony variance were made by PCA on standardized log-contrasts for the whole data set, before determining the within-colony variance component for each class of colonies for each PC and summing these variances to give the total. This is possible because the PC axes are not correlated. This was then repeated leaving out one colony at a time to generate the jack-knifed standard error (SE). These and subsequent calculations were performed using GenStat version 13 (VSN International Ltd, Hemel Hempstead, Herts, UK).

#### Hypothesis 2

##### An adult individual's profile becomes increasingly dissimilar from the colony profile during a period of isolation

This was tested by calculating the Euclidean distance in the space defined by standardized log-contrasts for each individual from the mean for its colony, based on individuals sampled directly from the colony and, therefore, not isolated. Distances were then analyzed using a GLM with terms for time isolated as a continuous effect, colony, and their interaction. Inspection of the data suggested that the main effect of isolation occurred before the first ant died (day 5). Therefore, we repeated the GLM excluding the nonisolated ants (time isolated = 0) and also with the continuous effect of time replaced by a two-level factor (nonisolated vs. isolated). Distances were not normally distributed and were dependent on the estimation of the colony mean from a small sample, so we tested significance by permuting the log-contrasts within colonies 1000 times, each time recalculating the colony means and distances. We then compared the variance ratios from the observed data with the null distribution obtained from the permutations.

#### Hypothesis 3

##### Isolated ants from different patrilines differ in their hydrocarbon profiles

This hypothesis was tested by conducting multivariate analyses of variance (MANOVAs) on the standardized log-contrasts with the patriline effect nested within the effect of colony and time isolated as a covariate (nonisolated ants were not included in these analyses).

We repeated the tests of hypotheses 2 and 3 for the isolated callows. Here, we used adults from the colony mound to determine the colony profile, but we did not include them when examining the effect of duration of isolation. Some callows died earlier than day 5 and we did not have a distinct class of nonisolated ants, so we conducted only one GLM analysis with a continuous effect of isolation. However, we also compared distances for adults from the colony with those for isolated callows.

We visualized the raw CHC profile data using triplots, because this method captures >90% of the cue diversity and facilitates a clear representation of profiles without data transformation.

## Results

### Hypothesis 1

#### Increased genetic diversity leads to an increased variation in the profile

Ongoing long-term genetic analysis of the study population (Haag-Liautard et al. 2009) has previously determined the mating frequency of 76 *F. exsecta* colonies, of which in this study 57 were singly mated and 12 doubly mated. Seven colonies were polygynous with 3–12 queens per nest, which were estimated from full sib-ships among the genotyped workers using the program Colony 2.0 (Wang 2004). The true number of queens within these colonies was likely to be much larger, because the workers genotyped represented only a fraction of the colony workforce.

**Figure 2:**
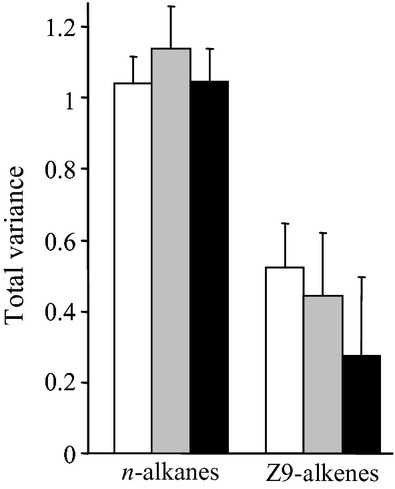
Comparison of total within-colony variance of *n*-alkanes and Z9-alkenes based on 688 individuals from 76 colonies with three different levels of genetic diversity, that is, singly mated (57 colonies, clear bars), doubly mated (12 colonies, gray bars), and polygynous (seven colonies, black bars). These estimates were made by conducting a PCA on the standardized log-contrasts for the whole data set, then finding the within-colony variance component for each class of colonies for each PC and summing these variances to yield the total. This was then repeated leaving out one colony at a time to generate the jack-knifed standard error.

A total of 688 high-quality total ion chromatograms were obtained from the 76 genotyped colonies. The total within-colony variance (relative to the population as a whole) for *n*-alkanes was higher than that for Z9-alkenes. There was no increase in total within-colony variance as genetic diversity increased for either *n*-alkanes or Z9-alkenes (Fig. 2), but instead a decrease was detected in the Z9-alkenes. Therefore, increased genetic diversity *did not* cause an increase in the variance of the colony profile.

### Hypothesis 2

#### An adult individual's profile becomes increasingly dissimilar from the colony profile during a period of isolation

**Figure 3:**
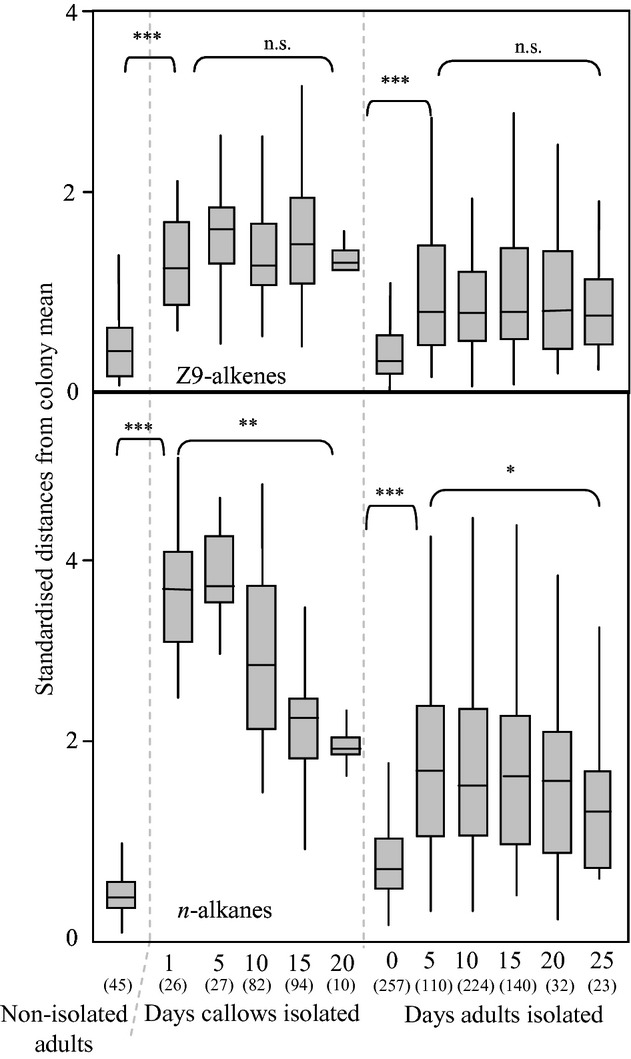
Effects of isolation period (days) on callows (left) and adults (right) for Z9-alkene (top) and *n*-alkane (bottom) profiles. Natural within-colony variation is represented by the distances of nonisolated individuals or adults isolated for zero days from their colony mean (*n* = 45 and 257). Sample sizes are given in parentheses. *P < 0.01, **P < 0.001, ***P < 0.0001.

**Figure 4:**
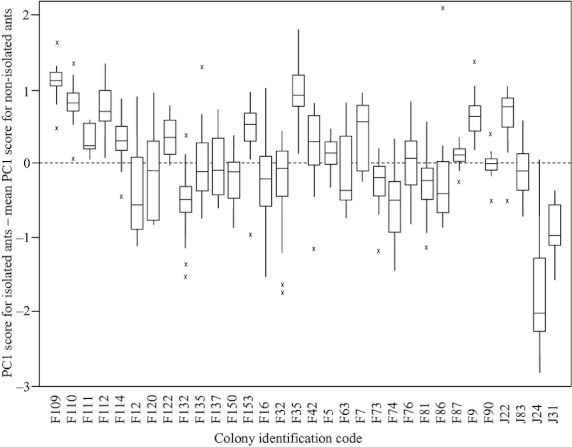
Box plots showing the distribution of PC1 scores (log-contrasts of Z9-alkenes that explains 75% of the variance) for 10–29 individual workers, minus their mean colony score, determined from 10 nonisolated ants. This shows that isolating workers resulted in a profile that differed from that of their parent colony in the majority of cases and that there was no consistent direction of change irrespective of colony chemotype.

Isolation of callows (*n* = 239) and adults (*n* = 529) resulted in their *n*-alkane and Z9-alkene profiles differing significantly from their original colonies (callows: *n*-alkanes F_1,223_ = 679.66, *P* < 0.001; Z9-alkenes F_1,223_ = 157.47, *P* < 0.001, adults: *n*-alkanes F_1,722_ = 412.62, *P* < 0.001; Z9-alkenes F_1,722_ = 364.57, *P* < 0.001) (Fig. 3). A significant change in the *n*-alkane and Z9-alkene profiles of isolated adults occurred within the first 5 days, but between day 5 and day 25, there was a trend toward decreasing distance for *n*-alkanes (F_1,722_ = 8.90, *P* = 0.007) and no significant change in distance for Z9-alkenes (F_1,722_ = 1.70, *P* = 0.62) (Fig. 3). The *n*-alkane and Z9-alkene profiles of newly emerged callows were already significantly further from the colony mean compared with workers in the colony (Fig. 3). These differences did not change significantly for the Z9-alkenes over the next 20 days of isolation (F_1,223_ = 1.34, *P* = 0.29). However, the *n*-alkane profile became more similar to the colony mean with the increasing isolation period (F_1,223_ = 327.69, *P* < 0.001), although after 20 days it remained more distant from the colony mean than did the Z9-alkenes. The mean profiles of isolated ants deviated from their colony means, but they drifted in no consistent direction (Fig. 4). Therefore, the period of isolation had no effect on an adult individual's Z9-alkene profile for callows or adults after the first 5 days because they did not become increasingly dissimilar from their colony profile.

### Hypothesis 3

#### Isolated ants from different patrilines differ in their hydrocarbon profiles

We compared CHC variation among isolated adult workers from two patrilines in each of nine colonies and callow workers in three colonies. To minimize any colony CHC transfer effects, all callows and adults were isolated for at least 5 days before determining their CHC profiles (see Fig. 3). A total of 283 individuals were isolated, before we determined their CHC profiles and genotyped them (Table 1). Four individuals were excluded from subsequent analysis, because their genotype and chemical profile did not match the colony they were collected from, indicating that these individuals were probably “drifters” from other nests. Their origin could be assigned to nearby colonies based on their genotype and chemotype. Two colonies were omitted from the comparison, because only one or zero sampled workers came from known patrilines. The observed paternity skew (mean = 0.630, SD = 0.155) matched that previously reported (Boomsma and Sundström 1998) for *F. exsecta* and other *Formica* spp.

**Table 1:** Distribution of patrilines in nine doubly mated *Formica exsecta* colonies sampled in September 2008 and three colonies sampled again in September 2009

Colony	Date	Patriline 1	Patriline 2	Skew	Total
J22	9/2008	7	3	0.58	10
J31	9/2008	7	14	0.56	21
F32	9/2008	9	7	0.51	16
F35	9/2008	18	2	0.82	20
F35	9/2009	26	8	0.64	34
F74	9/2008	12	9	0.51	21
F74	9/2009	19	15	0.51	34
F73	9/2008	17	3	0.75	20
F81	9/2008	15	11	0.51	26
F109*	9/2008	20	0	1.00	-
R10*	9/2008	1	8	0.80	-
F5	9/2008	14	15	0.50	29
F114	9/2008	11	8	0.51	19
F114	9/2009	25	8	0.63	33
				Mean = 0.63	283

* The two colonies were not analyzed due to their patriline skew. Paternity skew (the degree of prevalence of one patriline over the other) was calculated according to Pamilo (1993) and ranged from 0.5, where two males sired offspring equally, to 1, where a single male monopolized reproduction.

**Figure 5:**
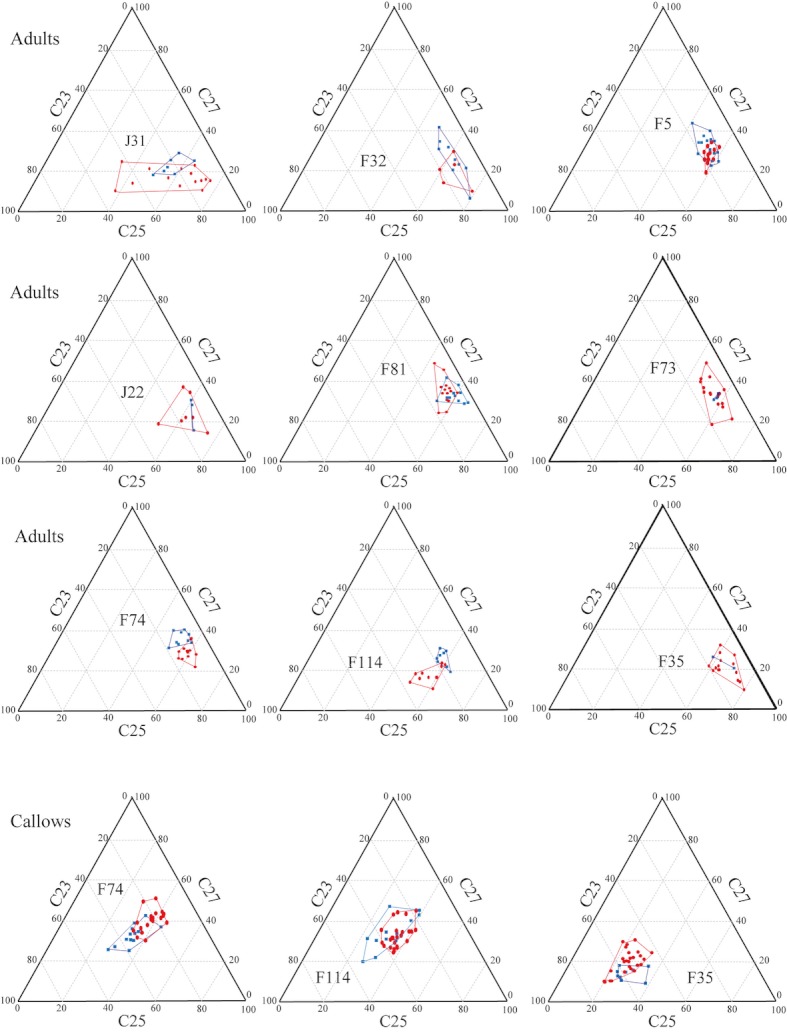
Proportions of the three most abundant *n*-alkanes of two patrilines (blue squares and red circles) for each of nine colonies. Polygons enclose each patriline. Callows or adult workers were isolated for a minimum of 5 days before CHC extraction. In these plots, a blend with 100% of one compound would appear at the relevant apex, a blend with only two compounds appears on the edge connected the relevant apices, and a three-component mixes appears in the body of the graph.

**Figure 6:**
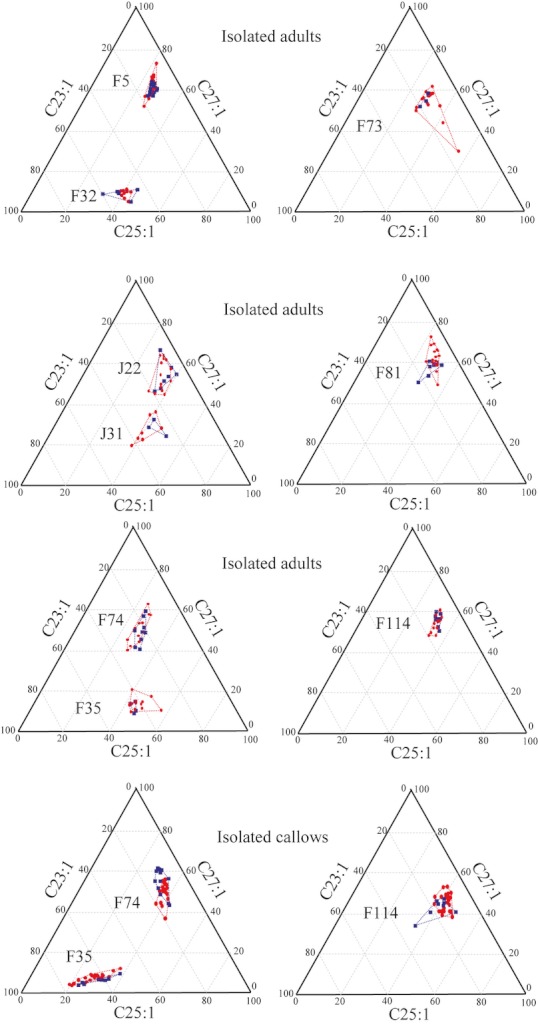
Triangular plots showing the proportions of the three most abundant Z9-alkenes of two patrilines (blue squares and red circles) for nine colonies. Colonies are distributed so colony profiles do not overlap. Polygons enclose each patriline. Callows or adult workers were isolated for a minimum of 5 days before CHC extraction. In these plots, a profile with 100% of one compound would lie at the relevant apex, a profile with only two compounds lies on the edge connected to the relevant apices, while a three compound profile lies within the body of the graph.

The three most abundant *n*-alkanes (Fig. 5) and Z9-alkenes (Fig. 6) typically accounted for >90% of the entire profile in *F. exsecta* and comparisons of CHC proportions using triangular plots showed that there was a great deal of overlap in CHC profiles between the two patrilines in each doubly mated colony, both for isolated callows and isolated adults. Nevertheless, patriline effects contributed a small, but significant, component of the CHC profile variance of isolated individuals in *n*-alkanes (MANOVA on standardized log-contrasts: for adults, Wilks' Λ = 0.625, approximate χ^2^_32_ = 396.9, *P* < 0.001; and callows, Wilks' Λ = 0.539, approximate χ^2^_12_ = 57.51, *P* < 0.001) and Z9-alkenes (adults, Wilks' Λ = 0.708, approximate χ^2^_36_ = 54.15, *P* < 0.027; callows, Wilks' Λ = 0.662, approximate χ^2^_12_ = 38.35, *P* < 0.001). Among-patriline variation was higher in callows compared with isolated adults and higher in *n*-alkanes compared with Z9-alkenes. The mean variance among isolated individuals due to patrilines (within colonies) was as follows: 20% in *n*-alkanes for callows (2–32% across the four log-contrasts); 5.8% in Z9-alkenes for callows (1–13%); 12% in *n*-alkanes for adults (8–17%); and 2.3% in Z9-alkenes for adults (0–6%). Therefore, isolated ants belonging to different patrilines had a weak, but significant, difference in both their Z9-alkene and *n*-alkane profiles.

## Discussion

This large-scale analysis of 76 *F. exsecta* colonies showed that increased genetic diversity, due to multiple mating or multiple queens (polygyny), did not increase the variation in the *n*-alkanes or Z9-alkenes among nestmates in a colony. This agrees with an independent analysis based on 15 colonies from this population (van Zweden et al. 2011). Two recent studies of the ants *F. fusca* (Helanterä et al. 2011) and *F. exsecta* (Martin et al. 2009b) also detected no increase in CHC profile variation associated with increased genetic diversity due to polygyny. These findings do not support the long-held assumption that colonies with greater genetic heterogeneity should have high recognition cue (CHC) variability, which is assumed to lead to lower discrimination abilities in polygynous societies (Hölldobler and Wilson 1990; Starks et al. 1998). Indeed, conspecific ant aggression (discrimination ability) has been shown to be correlated closely only with chemical (recognition cue) and not genetic distance (Martin et al. 2011). This may explain why many previous studies failed to detect the predicted differences in nestmate recognition between monogynous and polygynous colonies (e.g., Peeters 1988; Stuart 1988; Crosland 1990; Satoh and Hirota 2005; Rosset et al. 2007; Martin et al. 2009b). The decrease in the total variance among the Z9-alkenes supports a previous observation in *F. exsecta* of reduced, rather than increased, nestmate discrimination cue diversity in polygynous colonies (Martin et al. 2009b), although the reason for this effect is not known.

The absence of any increase in CHC profile variation with genetic diversity may be explained by the exchange of materials during grooming and trophallaxis. In this study, we investigated the CHC profiles of isolated adult ants and callows to control for the hypothetical homogenizing effect of this gestalt mechanism. This allowed us to elucidate the effects of patrilines by preventing any possible mixing interactions throughout a sustained period of isolation. As predicted by hypothesis 2, the profiles of isolated workers and callows deviated significantly from their colony profile, which as was previously shown in isolated ants (Lenoir et al. 2001) or those maintained in small queenless units (Yamaoka 1990). This deviation was already present in newly emerged callows. This is contrary to previous suggestions (Breed et al. 2004) that individuals lack a recognition profile at emergence, that is, they are a “blank slate”, although other CHCs such as *n*-alkanes must always be present to prevent desiccation. Profiles that deviated from their colony norms were apparent in adults during the first day of isolation, although we found little subsequent change in the CHC profiles in either group after being isolated for up to 20 days. The mean CHC profile of isolated ants (callows and adults) from a single colony also differed from the mean colony CHC profile in the majority of cases (Fig. 4). The gestalt hypothesis may explain this drift in the CHC profile of isolated ants, because it assumes that individual ants produce a genetically determined CHC profile that is mixed by grooming and trophallaxis to generate a uniform colony gestalt profile. However, this idea cannot explain the majority of our observations, since the profile generated by mixing the profiles of the isolated ants was often very different from that of their natal colony (Fig. 4).

The isolation of ants should allow individual ants to express their own genetically determined CHC profile and facilitate the discrimination of separate patrilines in doubly mated colonies. Isolation allowed us to detect significant patriline variation in both the *n*-alkanes and Z9-alkenes of individuals from doubly mated colonies. Van Zweden et al. (2011), studied the same *F. exsecta* population and found distinct CHC patriline profiles in nonisolated ants from only two of ten study colonies, and again only in the Z9-alkenes. By contrast, our study detected differences in all isolated ants, which is of crucial importance because it may facilitate the evaluation of genetic diversity at an appropriate developmental stage.

The consistent CHC profile of workers within colonies and the marked differences among colonies arose mainly from the shared environmental effects possibly mediated by the gestalt mechanism, whereas the small contribution of additive genetic variation was obscured within colonies. If the CHC profile (following isolation) was influenced by additive genetic variance (V_A_), the expected variation between patrilines within colonies would be 0.5 V_A_ whereas the variation between colonies would be 0.25 V_A_. This is because colonies with a single queen are mated to more than one male, and males are haploid. We assume no inbreeding. This partitioning of additive variance is the same as that for an X-linked trait in a normal diploid system. Thus, the low proportion of variance in the nestmate recognition component of the CHC profile, that is, the Z9-alkenes, accounted for by patrilines suggests a high nongenetic contribution (narrow sense heritability in the region of 5–10%). The variation among colonies remained high following isolation, so a substantial part of the nongenetic variation was colony-specific, that is, a common environmental or maternal effect that persists following isolation, whereas the paternal effect was small, unreliable, and would presumably be lost by gestalt mixing soon after the callows were adopted into the colony.

Plasticity in CHC profiles is well-documented in social insects, especially with respect to fertility and dominance signals (e.g., Dapporto et al. 2007; Peeters and Liebig 2009), but the hypothesis of a homogeneous colony CHC profile shared by all individuals and maintained by a gestalt process presents several problems. For example, if nepotism is costly to colonies, there should be selection against cues that allow discrimination of different patrilines. However, when a relatedness asymmetry exists in a colony, due to multiple mating or multiple queens, those colonies with high relatedness should favor female production, whereas those with low relatedness should favor male production (Boomsma and Grafen 1991). This predicted split sex ratio was found in *F. exsecta* colonies containing one or multiple queens, which suggests that workers might be able to detect patriline number in their colony (Sundström et al. 1996). In contrast, a more recent and much larger study found that the mating frequency did not affect the colony sex ratio in the same *F. exsecta* population (Vitikainen 2010), which supports the absence of a persistent patriline signal in this species, as suggested by both the current study and van Zweden et al. (2011).

Earlier research suggested that a patriline signal was present in the *n*-alkanes of *F. truncorum* ants (Boomsma et al. 2003) and *A. mellifera* honeybees (Arnold et al. 1996). Again, it is unclear how such a signal could persist in the presence of a putative gestalt mechanism that acts to homogenize the colony CHC profile, unless chemical cue production were high relative to the rate of mixing, which is unlikely due to the frequency of trophallactic interactions between nestmates. It was proposed that “selective mixing” of *n*-alkanes and other hydrocarbons (van Zweden et al. 2010) might allow certain signals to be preserved, although the gestalt mechanism and physical mixing properties of cuticular lipids (Gibbs 2002) make this unlikely. Furthermore, *n*-alkanes have been shown to be highly plastic and they even change with tasks in ants (Wagner et al. 2001; Martin and Drijfhout 2009a) and bees (Kather et al. 2011), and with the climate in flies (Savarit and Ferveur 2002). This would clearly negate the possibility of *n*-alkanes maintaining even a weak patriline signal. We detected a small but statistically significant patriline effect in both the Z9-alkenes and *n*-alkanes of isolated ants, but there was a large amount of overlap in the *n*-alkane (Fig. 5) and Z9-alkene (Fig. 6) profiles, which may suggest that a patriline recognition system would be highly error-prone even if it could be utilized at the level of isolated ants or, more likely, the callows. In *F. exsecta*, there is a close biochemical relationship between the production of *n*-alkanes and Z9-alkenes (Morgan 2010), so we often find statistically similar effects (this study; Martin et al. 2012), although only Z9-alkenes appear to affect behavior (Martin et al. 2008a, 2011).

It was suggested by van Zweden et al. (2011) that evaluations of patrilines might be possible at the egg or larval stage, because the CHC profile may be a more honest indicator of the genetic background of an individual prior to exposure to interactions with colony members and the homogenizing effects of the hypothetical gestalt mechanism. This is an interesting suggestion because we found that newly eclosed callow workers expressed highly diverse profiles that were significantly different from their prospective nestmates. Callows had previously been exposed to interaction with workers in the form of feeding prior to pupation, so the pupal period may have provided them with sufficient time to escape the colony profile and express their own profile, which was apparently more genetically, rather than environmentally, determined. We found support for this hypothesis in isolated adult workers, which lost their colony profile within 5 days. Further study of the profiles of isolated workers and callows may provide great insights into the genetic basis of the CHC profile and also elucidate whether processes that are expected to require some degree of genetic kin differentiation might possibly operate at the level of the brood, that is, eggs, larvae, and pupae.

The presence or absence of a patriline signal among social insects is a contentious area and different groups have their own preferred hypotheses and statistical approaches, which were considered after reviewer comments regarding the current manuscript. Traditionally, compositional data analysis based on principal components has been applied to complete or partial hydrocarbon data sets, typically without characterizing specific molecules, to test for differences between various groups in multidimensional space. However, this approach has numerous disadvantages, including strong variance contributions from low-abundance components, multicollinearity (Martin and Drijfhout 2009c), and the failure to detect sample contamination. Recently, Charpentier et al. (2012) highlighted a litany of errors in mammalian pheromone studies due to the “blind” analysis of chemical data, before advocating that chemical ecologists should first identify individual chemical components in all future studies to validate their biological relevance. We have adopted this approach of identifying specific molecules in our study species and we only analyzed those known compounds which have been strongly implicated in nestmate recognition. A reviewer of the current study suggested that we should combine the alkane and alkene data, but it is difficult to see how combining the two chemical classes could provide any additional information. We detected a weak patriline effect in the alkenes when the two classes of hydrocarbon were analyzed separately, and this effect would contribute a smaller proportion of the variance and may be undetectable if we pooled both classes of hydrocarbons. Clearly, a finer level of analysis based on molecules with known effects is a more rational analytical approach. Furthermore, the Z9-alkenes appear to be used for species (Martin et al. 2009b) and nestmate recognition (Martin et al. 2008a), so we anticipated that a putative patriline signal might be nested within a nestmate signal, although at a finer scale. It is parsimonious to expect that recognition will be mediated by the same set of compounds. A reviewer of this study also proposed the alternative hypothesis that a separate set of compounds might mediate patriline recognition. This is not only unparsimonious but also unlikely because the existence of such a system would facilitate kin discrimination and increase the risk of nepotism among social insects, whereas it has only been claimed very rarely in empirical studies.

The current study supports the hypothesis that interactions between nestmates at the level of workers from other colonies are not based on any evaluation of genetic kinship. Instead, colony recognition (nestmate) profiles are unique to colonies and they may be a result of the hypothetical gestalt mechanism. However, it is possible that certain interactions within the nest could be mediated by an evaluation of genetic kinship because patriline effects were detectable in callow workers, and presumably pupae, larvae, and eggs. But, even here we must note that the mechanism underlying such a comparative act remains obscure, because if an adult worker made such an inspection it would only be able to make reference to its own innate phenotype as a comparator, which matches the CHC profile of the entire colony. Clearly, this would eliminate the possibility of nepotism. However, it may still be possible to perform relative comparative evaluations among the brood items, which could provide information on the genetic diversity present in the colony. Such an evaluation could facilitate the adjustment of colony kin structure to yield the split sex ratios that have frequently been reported in *Formica* species (Kümmerli and Keller 2009).

Thus, we have shown that the relative contributions of genetic and environmental factors to nestmate recognition could vary depending on the context. The diversity of CHC profiles detected in isolated ants and callows was significantly affected by their patrilines, whereas adult workers in colonies showed no detectable patriline effects. We are hopeful that further study of isolated workers and callows in this model system may elucidate the elusive source of genetic recognition signals in ants.
